# Characterization of pneumococcal Ser/Thr protein phosphatase *phpP* mutant and identification of a novel PhpP substrate, putative RNA binding protein Jag

**DOI:** 10.1186/s12866-016-0865-6

**Published:** 2016-10-24

**Authors:** Aleš Ulrych, Nela Holečková, Jana Goldová, Linda Doubravová, Oldřich Benada, Olga Kofroňová, Petr Halada, Pavel Branny

**Affiliations:** Institute of Microbiology, v.v.i., Academy of Sciences of the Czech Republic, Vídeňská 1083, 142 20 Prague, Czech Republic

**Keywords:** Signal transduction, Protein phosphatase, Protein kinase, Cell division, *Streptococcus*, Phosphorylation, Jag

## Abstract

**Background:**

Reversible protein phosphorylation catalyzed by protein kinases and phosphatases is the primary mechanism for signal transduction in all living organisms. *Streptococcus pneumoniae* encodes a single Ser/Thr protein kinase, StkP, which plays a role in virulence, stress resistance and the regulation of cell wall synthesis and cell division. However, the role of its cognate phosphatase, PhpP, is not well defined.

**Results:**

Here, we report the successful construction of a Δ*phpP* mutant in the unencapsulated *S. pneumoniae* Rx1 strain and the characterization of its phenotype. We demonstrate that PhpP negatively controls the level of protein phosphorylation in *S. pneumoniae* both by direct dephosphorylation of target proteins and by dephosphorylation of its cognate kinase, StkP. Catalytic inactivation or absence of PhpP resulted in the hyperphosphorylation of StkP substrates and specific phenotypic changes, including sensitivity to environmental stresses and competence deficiency. The morphology of the Δ*phpP* cells resembled the StkP overexpression phenotype and conversely*,* overexpression of PhpP resulted in cell elongation mimicking the *stkP* null phenotype. Proteomic analysis of the *phpP* knock-out strain permitted identification of a novel StkP/PhpP substrate, Spr1851, a putative RNA-binding protein homologous to Jag. Here, we show that pneumococcal Jag is phosphorylated on Thr89. Inactivation of *jag* confers a phenotype similar to the *phpP* mutant strain.

**Conclusions:**

Our results suggest that PhpP and StkP cooperatively regulate cell division of *S. pneumoniae* and phosphorylate putative RNA binding protein Jag.

**Electronic supplementary material:**

The online version of this article (doi:10.1186/s12866-016-0865-6) contains supplementary material, which is available to authorized users.

## Background

Signal transduction via protein phosphorylation is one of the basic mechanisms that modulate numerous cellular processes in both prokaryotes and eukaryotes. Signal transduction in prokaryotes has been considered to occur primarily by two-component systems consisting of a histidine protein kinase and its cognate response regulator [1]. However, studies published in the last two decades have clearly demonstrated that this paradigm requires modification. Eukaryotic-type Ser/Thr protein kinases (ESTKs) as well as Ser/Thr phosphatases (ESTPs) operate in various bacterial species in parallel or overlapping signaling networks and regulate various cellular functions [2]. A distinct group of ESTKs which regulate cell cycle and cell division in many Gram-positive bacteria are conserved transmembrane proteins with a cytoplasmic kinase domain and repeated PASTA (penicillin-binding protein and Ser/Thr kinase-associated) domains in their extracellular region [2–6].

ESTKs are often co-expressed with their cognate phosphatases which are necessary for regulation of ESTK activity and quenching of signaling cascades; however, their physiological function in bacteria is still poorly understood. The ESTPs associated with PASTA-possessing ESTKs are Mg^2+^- or Mn^2+^-dependent enzymes of the PPM family of Ser/Thr phosphatases, which share homology with the eukaryotic PP2C phosphatase [7]. Unlike ESTKs, only a few cognate ESTPs have been studied in detail, in part because several of them have been reported to be essential [8–10]. However, other detailed studies have demonstrated that knock-out mutants of phosphatase genes are viable and that ESTPs play a role in virulence, cell wall metabolism and cell segregation [11–16].

*S. pneumoniae* encodes a single PASTA-containing ESTK named StkP and a co-transcribed phosphatase, PhpP [8, 17]. Unlike PhpP, StkP has been extensively studied in past years, and its pleiotropic function in the regulation of different cellular processes has been described. StkP is a virulence determinant that is important for lung infection and bloodstream invasion in vivo and regulates pilus expression and bacterial adherence in vitro [8, 18]. StkP is essential for the resistance of *S. pneumoniae* to various stress conditions and competence development. Microarray analysis has revealed that StkP affects the transcription of a set of genes involved in cell wall metabolism, pyrimidine biosynthesis, DNA repair, iron uptake and oxidative stress response [8, 19]. StkP localizes to the division sites and plays important role in the regulation of cell division [20–22]. Cells with *stkP* mutations demonstrated disrupted cell wall synthesis and displayed elongated morphologies with multiple, often unconstricted, cell division septa, which suggest that StkP coordinates cell wall synthesis with cell division and thus helps pneumococcus to achieve its characteristic ovoid shape. Consistent with its role in cell division, StkP was found to phosphorylate several proteins involved in cell wall synthesis and cell division. The cell division proteins DivIVA [21, 23], LocZ (named also MapZ) [23–25] and the phosphoglucosamine mutase GlmM [17] are phosphorylated by StkP in vitro and in vivo. The cell division proteins FtsZ [22] and FtsA [20] and the cell wall biosynthesis enzyme MurC [26] are substrates of StkP in vitro; however, their phosphorylation by StkP in vivo has not been confirmed.

StkP is dephosphorylated by the cognate phosphatase PhpP, which is a PP2C-type Mn^2+^-dependent enzyme. The PhpP catalytic domain contains 11 conserved signature motifs [27], and mutations of the highly conserved residues D192 and D231, which have been implicated in metal binding, completely abolish PhpP activity in vitro [17]. GFP-PhpP fusion protein is localized in the cytoplasm; however, the protein is often enriched in the midcell. The localization of PhpP to cell division sites depends on the presence of active StkP, indicating that both enzymes form a signaling couple in vivo [20]. Previously, *phpP* was reported to be essential for the viability of the unencapsulated Rx1 and R800 strains [10, 21]. According to global analysis performed by Thanassi et al. [28], both *phpP* and *stkP* genes were found to be essential; however, in the other global studies, *phpP* was not recognized as an essential gene [29, 30]. A recent report generated nonpolar markerless *phpP* knock-out mutants in two encapsulated pathogenic strains, *S. pneumoniae* D39 and 6A, indicating that PhpP is dispensable for pneumococcal survival [11]. Characterization of these mutants demonstrated the strain-specific role of PhpP in cell wall biosynthesis, adherence and biofilm formation. The StkP/PhpP signaling couple has been demonstrated to regulate the two-component system HK06/RR06, which modulates the expression of a major pneumococcal adhesin, CbpA [11].

In the present study, we show that the unencapsulated Rx1 *phpP* knock-out strain is viable. The morphology of both, the unencapsulated *phpP* null mutant and the *phpP* overexpression strain, clearly demonstrated that PhpP participates in the regulation of cell division and has an opposite regulatory effect to that of StkP. Our data suggest that PhpP modulates the level of protein phosphorylation in vivo both, through direct dephosphorylation of target proteins and dephosphorylation of its cognate kinase, StkP, resulting in coordination of cell wall synthesis and division in *S. pneumoniae*. Proteomic analysis of the Δ*phpP* strain revealed a novel StkP/PhpP substrate, Spr1851, a putative RNA-binding protein homologous to Jag protein of *B. subtilis* [31].

## Results and discussion

### PhpP is not essential for pneumococcal survival and catalyzes dephosphorylation of StkP and its substrates

**Fig. 1:**
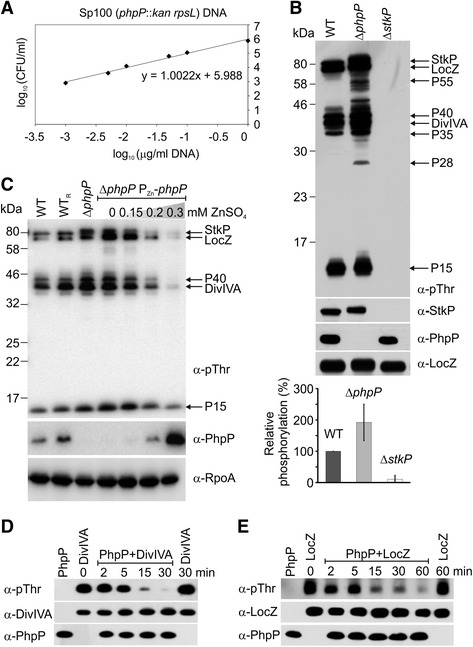
PhpP regulates phosphorylation of StkP and its substrates. **a** Kinetics of transfer of *phpP* mutation. WT strain was transformed by Sp100 genomic DNA carrying Janus cassette inserted into *phpP* gene (*phpP::kan rpsL*). Number of kanamycin resistant transformants was plotted as a function of genomic DNA concentration in logarithmic scale. Transfer of tested marker follows linear kinetics with slope of line equal to 1 (y = ax + b, a = slope). **b** Phosphorylation profile of Δ*phpP* mutant. Membrane fraction from *S. pneumoniae* WT (Sp1), Δ*phpP* (Sp113) and Δ*stkP* (Sp10) was isolated and 30 μg of proteins was subjected to SDS-PAGE and immunoblotted with anti-phospho-threonine antibody (α-pThr) to detect phosphorylated proteins. The level of PhpP and StkP proteins was detected with anti-PhpP (α-PhpP) and anti-StkP antibody (α-StkP). Immunodetection of membrane protein LocZ was used as loading control. Arrows indicate position of StkP and its known (LocZ, DivIVA) and unknown (P15, P28, P35, P40, P55) substrates. Relative phosphorylation values represent mean ± SD. **c** Complementation of *phpP* mutation. Comparison of phosphoprotein pattern of wild type strain WT (Sp1), reverted wild type strain WT_R_ (Sp222), Δ*phpP* strain (Sp113) and complementation strain Δ*phpP* P_Zn_-*phpP* (Sp120) cultivated in C + Y medium in the presence or absence of inducer (ZnSO_4_). Total protein lysates (30 μg) were subjected to SDS-PAGE and immunoblotted with α-pThr antibody to detect phosphorylated proteins. The total amount of PhpP was detected using α-PhpP antibody. Immunodetection of α-subunit of RNA polymerase (α-RpoA) was used as loading control of total cell lysate. Arrows indicate position of StkP substrates. **d** PhpP dephosphorylates DivIVA. **e** PhpP dephosphorylates LocZ. Purified DivIVA-Flag and Flag-LocZ were incubated with His-PhpP in vitro and reaction was stopped at indicated times. Samples were subjected to SDS-PAGE and immunoblotted with α-pThr antibody to visualize dephosphorylation in time. PhpP, LocZ and DivIVA were detected with specific antibodies as described above. To exclude the spontaneous decay, phosphorylated form of both proteins, DivIVA-Flag and Flag-LocZ, was incubated for 30 and 60 min, respectively, in phosphatase reaction buffer without addition of His-PhpP

Although *phpP* was reported to be essential in an *stkP* ^+^ genetic background [10], a nonpolar markerless *phpP* knock-out was generated in two encapsulated *S. pneumoniae* strains using the Janus cassette-based two-step negative selection strategy [11]. We attempted to use the same strategy to knock out *phpP* in the unencapsulated Rx1 strain as described in the *Methods* *.* We obtained viable Δ*phpP* transformants and characterized them further (see below). To exclude the possibility that the Δ*phpP* strain might carry an unlinked extragenic suppressor of the potentially lethal effect of loss of PhpP, the dose-response pattern for Δ*phpP* versus a wild type (WT) backcross was determined [32, 33]. Transformation by a single marker in pneumococcus displays a linear dependence on the dose of donor DNA (slope of regression curve equal to 1), whereas less efficient co-transformation by two markers follows a quadratic dependence on donor DNA dose (slope of regression curve equal to 2). Transfer of the Δ*phpP* mutation followed first order kinetics, and, therefore, viability of the Δ*phpP* strain does not depend on an extragenic suppressor mutation (Fig. 1a). In addition, the sequence of the neighboring genes *spr1579* and *stkP* was verified for the absence of mutation by DNA sequencing. The contradictory results reporting the essentiality [10, 21, 28] and non-essentiality [11, 29, 30] of the *phpP* gene may result from the different methods used for gene inactivation or from the genetic variability of the pneumococcal strains used. As reviewed in Massidda et al*.* [34], the genome of *S. pneumoniae* is very dynamic, and the number of genes found to be conditionally essential is dependent on the genetic background or the presence of capsule.

Using specific anti-PhpP (α-PhpP) and anti-StkP (α-StkP) antibodies, we confirmed that PhpP was deleted from the genome of the Δ*phpP* strain (Sp113), while the expression level of StkP was similar in both the Δ*phpP* and wild type strain (Fig. 1b). To evaluate the level of protein phosphorylation, we performed immunodetection with an anti-phospho-threonine (α-pThr) antibody. Thr phosphorylation in *S. pneumoniae* is largely dependent on StkP, and the majority of its substrates are membrane or membrane-associated proteins [23]. As previously reported, no Thr phosphorylated proteins were detected in the Δ*stkP* mutant (Sp10) (Fig. 1b). Immunodetection of phosphoproteins in the Δ*phpP* membrane fraction revealed a pattern similar to the StkP-dependent phosphoproteome [23]; however, we observed an increase in signal intensity corresponding to 192 ± 58.4 % of the wild type, indicating hyperphosphorylation of StkP substrates, including StkP itself (Fig. 1b). These data indicate that PhpP negatively regulates phosphorylation of StkP and its substrates.

To verify that the observed phosphorylation profile was the result of the *phpP* deletion, we constructed two complementation strains. First, we reverted the Δ*phpP* mutation back to the wild type genotype by transforming the wild type allele of the *phpP* gene into the Δ*phpP* strain (strain Sp222). The second complementation strain was prepared using ectopic expression of *phpP* under the control of a zinc-inducible promoter, P_*czcD*_ (P_Zn_) [35]. *phpP* was cloned into the pJWV25 plasmid under the control of the P_Zn_ promoter and was inserted by double cross-over into the dispensable *bga* locus on the chromosome of the Δ*phpP* strain. The resulting strain, Sp120 (Δ*phpP bga:*:P_Zn_-*phpP*), was cultivated in the presence of different concentrations of ZnSO_4_, and the expression of PhpP and the level of protein phosphorylation was monitored by immunoblotting. When expression of *phpP* was induced by addition of 0.2 mM ZnSO_4_, we observed expression of PhpP and a phosphorylation signal intensity that correlated with the wild type (Fig. 1c, lane 6). Addition of 0.3 mM ZnSO_4_ resulted in PhpP overexpression and a strong decrease in Thr phosphorylation (Fig. 1c, lane 7). The reverted strain WT_R_ (Sp222) showed Thr phosphorylation levels that were indistinguishable from the wild type strain (Fig. 1c, lane 2). These results demonstrate that complementation restores the wild type phosphorylation profile.

To demonstrate that PhpP directly dephosphorylates StkP substrates and not solely StkP, thus decreasing its activity, we performed in vitro dephosphorylation assays. We prepared pneumococcal strains Sp188 and Sp174 expressing the known StkP substrates LocZ and DivIVA, respectively, tagged with Flag-tag and isolated phosphorylated Flag-LocZ and DivIVA-Flag proteins from pneumococcal cell lysates. Purified proteins were incubated with recombinant His-PhpP as described in *Methods*, and protein dephosphorylation was monitored using immunodetection with the α-pThr antibody. As shown in Fig. 1d and e, the phosphorylation of DivIVA and LocZ significantly decreased with time, demonstrating that PhpP directly catalyzes dephosphorylation of both StkP substrates.

### The *phpP* knock-out strain is sensitive to elevated temperature and oxidative stress

**Fig. 2:**
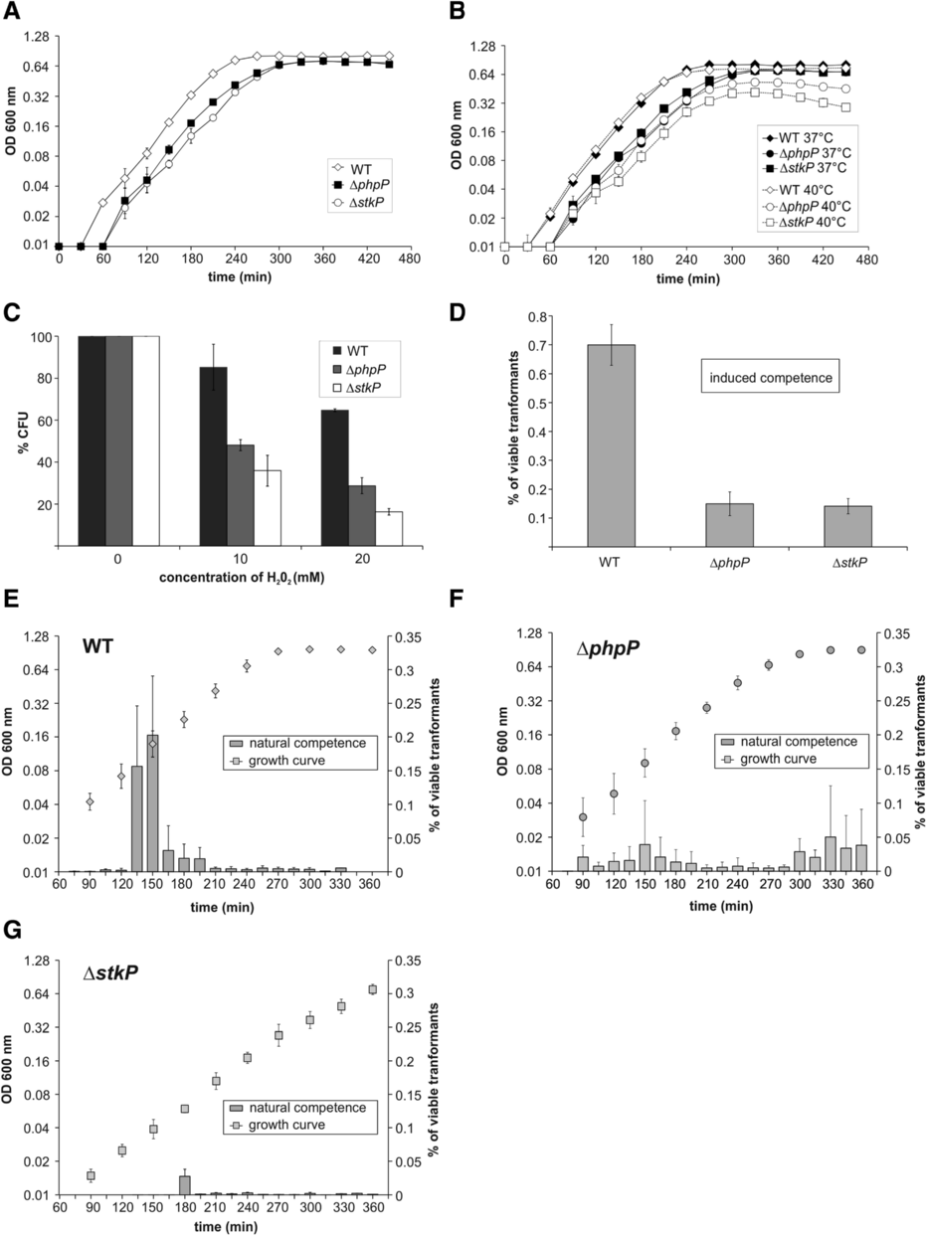
Phenotypic characterization of *phpP* knock-out strain. **a** Growth curves of WT (Sp1), Δ*stkP* (Sp10), and Δ*phpP* (Sp113) strains cultivated statically at 37 °C in TSB medium. **b** Heat stress. Growth curves of the WT and both mutant strains in TSB medium were measured at 37 and 40 °C. **c** Oxidative stress. The liquid cultures of the WT, Δ*stkP* and Δ*phpP* strain were exposed to different concentration of H_2_O_2_ and aliquots of cultures were plated to evaluate survival upon oxidative stress. Number of CFU (colony forming units) at non-stress conditions (0 mM H_2_O_2_) were set as 100 %. **d** Induced competence. Transformation efficiency of WT, Δ*phpP* and Δ*stkP* strain grown in BHI medium upon addition of CSP. **e**, **f**, **g** Natural competence. Natural competence is expressed as % of viable transformants of WT (**e**), Δ*phpP* (**f**) and Δ*stkP* (**g**) strain during cultivation in BHI medium (OD_600nm_) without addition of CSP. All data shown represent mean ± SD for three independent experiments. Where error bars are not shown, the SD was within the size of the symbol. All growth curves are plotted in semi logarithmic scale

The *stkP* null mutant has an altered growth rate, and it is sensitive to environmental stresses [19], which highlights the importance of StkP in the resistance of pneumococcus to hostile environmental conditions in the host. The growth rate of the *phpP* knock-out strain was reduced in TSB medium (38 min doubling time) compared to the wild type strain (31 min doubling time). In addition, the mutant strain had a significantly prolonged lag phase and reached a lower final optical density (Fig. 2a), similar to the Δ*stkP* mutant strain. Further we examined the growth of the Δ*phpP* mutant in response to heat stress, osmotic stress and pH variation, as well as its viability after exposure to H_2_O_2_. The Δ*phpP*, Δ*stkP* and the wild type strains were inoculated in liquid medium and cultivated as described in detail in the *Methods* *.* Our experiments showed that unlike StkP, PhpP did not significantly affect the sensitivity to osmotic stress induced by high salt concentration or the tolerance to acidic or alkaline pH (data not shown). However, PhpP was important for normal growth at elevated temperatures: the doubling time and final density achieved were significantly affected when the Δ*phpP* strain was grown at 40 °C (Fig. 2b). In addition, we tested the resistance of the mutant strain to oxidative damage. When exposed to varying concentrations of H_2_O_2_, the Δ*phpP* strain, similar to the Δ*stkP* strain, displayed a lower survival rate than the wild type strain, indicating increased sensitivity to oxidative stress (Fig. 2c). In summary, the phenotype of the unencapsulated Rx1 derived Δ*phpP* mutant differs from the encapsulated strain 6A which displayed retarded growth under all stress conditions tested but also differs from the strain D39 which was affected only in the high-salt stress conditions [11]. These results suggest that genetic background significantly affects the demonstration of *phpP* mutation although we cannot exclude the role of polysacharide capsule itself. Considering that the Rx1 derived *stkP* mutant strain was sensitive to elevated temperature, acidic pH, osmotic and oxidative stress [19], we did not confirm the opposite effect of PhpP. Our data suggest that unbalanced activity of both, PhpP and StkP, is critical for bacterial physiology, and the adaptive response to environmental stress is not cooperatively regulated by the PhpP/StkP signaling couple.

### The Δ*phpP* strain displays decreased competence for genetic transformation

Competence for genetic transformation is powerful mechanism for generating genetic diversity and acquiring antibiotic resistance. Natural competence in *S. pneumoniae* is a transient event regulated by a quorum-sensing system and occurs via a peptide pheromone signal (e.g., competence-stimulating peptide (CSP)). Previous studies demonstrated the importance of StkP for competence development [10, 19]. Here, we tested the capability of the Δ*phpP* mutant strain to develop natural and induced competence in conditions optimal for competence development [36]. Induced competence was defined as the transformation efficiency in response to the addition of synthetic CSP. Similar to the Δ*stkP* strain, the *phpP* null mutant strain weakly developed induced competence, and the transformation efficiency was low compared to wild type (Fig. 2d). Natural competence was defined as the transformation efficiency in the absence of added CSP and was monitored during the growth. As shown in Fig. 2e, that wild type strain developed natural competence during the early exponential phase of growth (OD_600_ 0.08–0.16), which is observed as a peak in viable transformants obtained by transformation with control DNA. On the other hand, two low peaks of competence, one during the exponential phase and the second upon the entry into the stationary phase, were detected during growth of the *phpP* knock-out strain (Fig. 2f). However, the transformation efficiency was about fivefold lower than that observed for the wild type, and therefore, the strain is competence deficient, similar to the Δ*stkP* strain (Fig. 2 g). In the Δ*stkP strain* the reduced transformation efficiency may be the result of a weak induction of DNA uptake and processing genes [19]. However, the molecular mechanism responsible for the transformation deficiency in the Δ*stkP* mutant remains unclear. To date, none of the proteins that play a direct role in competence development have been identified as a substrate of StkP. Our results suggest that StkP and PhpP do not function as antagonists in the control of competence regulation. Therefore, we cannot exclude that competence deficiency of both mutants is an indirect consequence of the pleiotropic effects of *phpP* and *stkP* mutations on pneumococcal physiology.

### PhpP regulates cell division in pneumococcus

**Fig. 3:**
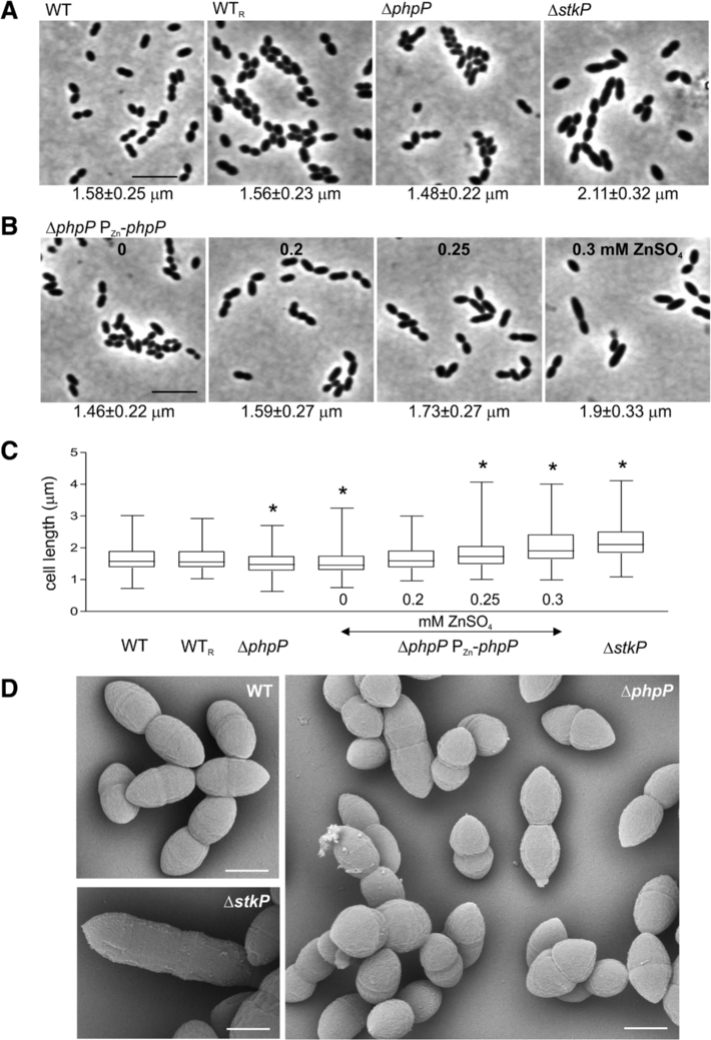
PhpP is involved in regulation of cell division in *S. pneumoniae*. **a** Morphology and cell length analysis of WT strain (Sp1), reverted strain WT_R_ (Sp222) and Δ*phpP* (Sp113) or Δ*stkP* (Sp10) strains grown in C + Y medium to mid-exponential phase. **b** Cell length depends on expression of PhpP in complementation strain Δ*phpP* P_Zn_-*phpP* (Sp120). Micrographs of complementation strain cultivated in the presence of 0, 0.2, 0.25 and 0.3 mM ZnSO_4_ in C + Y medium. Cell length in panel A and B is expressed as a median value ± MAD (*n* = 300). Bar 5 μm. **c** Cell length analysis. Cell length parameters measured with MicrobeTracker software were analyzed and plotted in box-and-whiskers graph. Mann-Whitney *U* test: * cell length of mutant strain is significantly different from that of the WT strain *P* < 0.0001. 300 cells were scored per sample. **d** Scanning electron microscopy of WT strain (Sp1), Δ*stkP* strain (Sp10) and Δ*phpP* strain (Sp113) cultivated in TSB medium. Magnifications are the same for all panels. Bar 0.5 μm. a-d: Representative data for three independent experiments are shown

The newly established role of StkP in cell division prompted us to investigate the morphology of the *phpP* knock-out strain and the potential role of PhpP in the regulation of cell division. Although no morphologic changes were observed in the encapsulated Δ*phpP* mutants [11], phase contrast microscopy of the Rx1 derived Δ*phpP* strain revealed that the phenotype differed from that of the wild type strain. Cell size measurement using the automated MicrobeTracker software confirmed that the Δ*phpP* cells were significantly smaller (median cell length 1.48 ± 0.22 μm; median cell width 0.64 ± 0.08 μm) than the wild type cells (median cell length 1.58 ± 0.25 μm; median cell width 0.66 ± 0.04 μm) (Fig. 3a, c) and showed phenotype similar to the StkP-overexpressing strain. To demonstrate that the observed phenotype was the result of the *phpP* deletion, we analyzed the reverted strain WT_R_ (Sp222) and the complementation strain Δ*phpP* P_Zn_-*phpP* (Sp120). Cell morphology (Fig. 3a) and cell size (Fig. 3c) of the reverted strain were not different from the wild type strain. The analysis of the complementation strain Sp120 cultivated in the presence of a growing concentration of the inducer (ZnSO_4_) demonstrated that increasing expression of PhpP correlated with the decrease in protein phosphorylation (Fig. 1c) and an increase in cell length (Fig. 3b, c). In the presence of 0.3 mM ZnSO_4_, the median cell length reached 1.9 ± 0.33 μm (Fig. 3b, c), clearly indicating that overexpression of PhpP results in a phenotype similar to that observed with the StkP-depleted strain (strain Sp10, median cell length 2.11 ± 0.32 μm) (Fig. 3a, c).

To obtain further insight into the morphological changes induced by inactivation of *phpP*, we analyzed the mutant strain Sp113 by electron microscopy. Figure 3d shows a scanning electron microscopy image of the wild type (Sp1) compared with the Δ*phpP* (Sp113) and Δ*stkP* (Sp10) strains. As reported previously, the Δ*stkP* strain produces long cells with unconstricted septa. On the other hand, we observed cell size heterogeneity in Δ*phpP* mutant cells, with numerous smaller cells; however, their shape appeared to be normal in general. Additionally, transmission electron microscopy did not reveal significant abnormalities (thicker cell walls) associated with the loss of *phpP* (data not shown) which were observed in the encapsulated Δ*phpP* strain [11].

Analysis of 600 cells showed that 24.2 % of the Δ*phpP* cells formed chains longer than 4 cells in contrast with 2.5 % of chaining cells in the wild type. However, we did not observe aggregation and abnormally long chains which were detected in the encapsulated mutant strains [11]. Regulation of chain length in streptococci depends on wall-associated autolytic activity. Therefore we tested the expression of genes encoding the peptidoglycan hydrolases *pcsB*, *lytA* and *lytB*, which may affect cell separation, using qRT-PCR, but we did not detect any differences in transcript levels in the Δ*phpP* and wild type strain (data not shown); thus, the reason for the increased chain formation remains unknown.

**Fig. 4:**
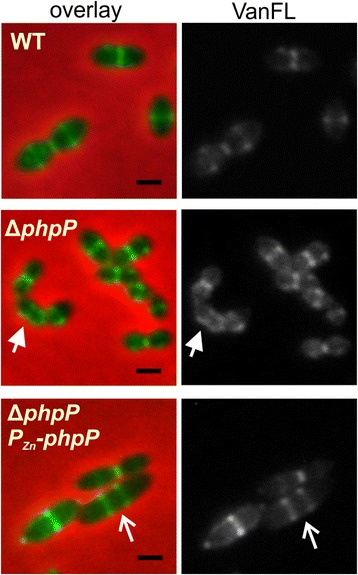
Cell wall synthesis in Δ*phpP* mutant. Nascent peptidoglycan synthesis sites in live pneumococcal cells of WT (Sp1), Δ*phpP* (Sp113) strain and overexpression strain Δ*phpP* P_Zn_-*phpP* (Sp120) were stained by fluorescently labelled vancomycin (Van-FL). Overexpression of PhpP in strain Δ*phpP* P_Zn_-*phpP* was achieved by addition of 0.3 mM ZnSO_4_ into C + Y medium. Full arrow shows cell with disturbed nascent peptidoglycan synthesis. Open arrow indicate multiple unconstricted cell division septa. Van-FL signal and its overlay with phase contrast images are shown. Scale bars 1 μm

To characterize the role of PhpP in cell division in more detail, we investigated the localization of nascent PG synthesis sites in live Δ*phpP* cells (Sp113) stained with fluorescently labeled vancomycin (Van-FL), a marker of nascent peptidoglycan synthesis (PG) (Fig. 4). Labeling was observed predominantly at current and future cell division sites in the mutant cells, a pattern similar to that observed in the wild type cells. However, 4.5 % of mutant cells (58/1300) showed disturbed Van-FL labeling (Fig. 4) indicating that minority of cells display perturbed cell wall synthesis. When we induced overexpression of PhpP in complementation strain Sp120 (Δ*phpP* P_Zn_-*phpP*) by the addition of 0.3 mM ZnSO_4_, we observed significant elongation of cells, and Van-FL staining revealed that the cells often contained multiple unconstricted division septa, which is a distinct feature of *stkP*-depleted cells (Fig. 4). Further we investigated localization of cell division proteins LocZ/MapZ, FtsA and DivIVA but we did not find significant differences between mutant and wild type cells (data not shown).

Our data clearly show that PhpP plays an opposing role to StkP in regulation of cell division which was not recognized in the previous study by Agarwal et al. [11]. We hypothesize that the morphological differences between Δ*phpP* mutants derived from different strains may be largely caused by the presence or absence of capsule. However, the cell division defect caused by the depletion of *phpP* is less severe than the abnormalities observed either in the absence of StkP or in the presence of the excess of PhpP suggesting that hyperphosphorylation of StkP substrates is better tolerated than the absence of phosphorylation.

### Conserved residues D192 and D231 are essential for PhpP activity *in vivo*

**Fig. 5:**
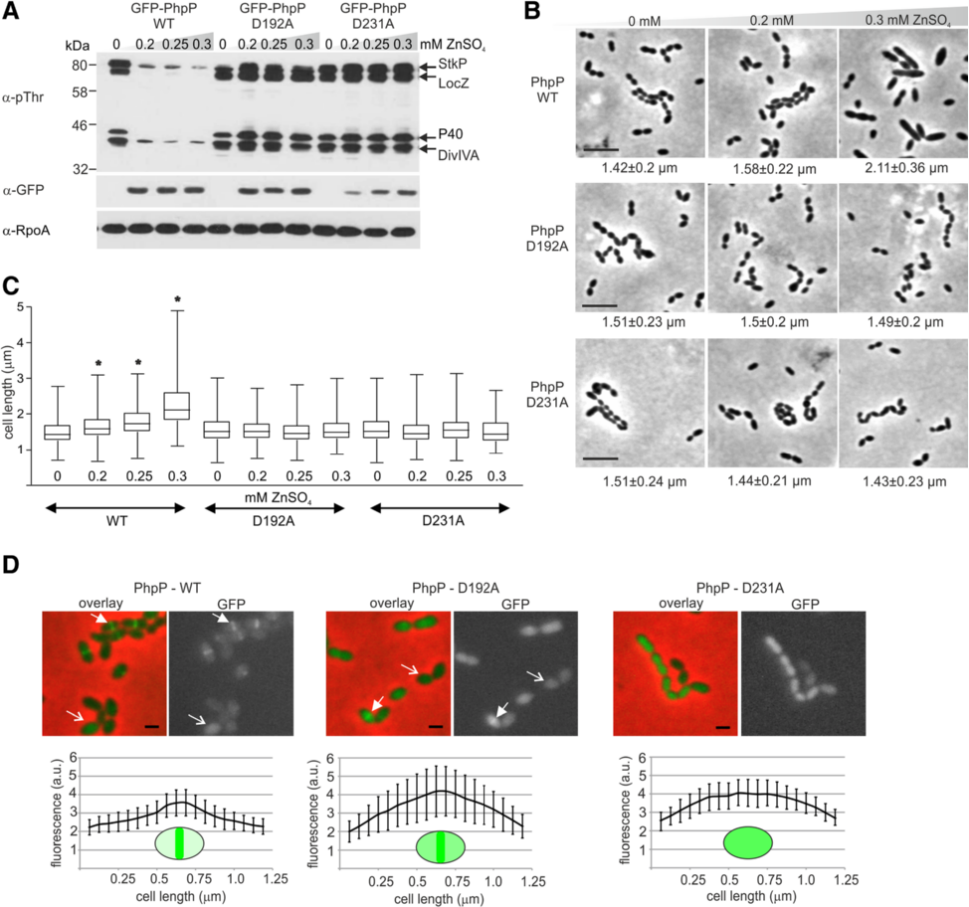
D192 and D231 are essential for PhpP catalytic activity *in vivo*. **a** Phosphorylation pattern after induction of expression of GFP-PhpP-WT (Sp140), GFP-PhpP-D192A (Sp292) and GFP-PhpP-D231A (Sp293) in Δ*phpP* genetic background. Total cell lysates from cultures grown in C + Y medium in the presence or absence of ZnSO_4_ were separated by SDS-PAGE and immunoblotted with α-pThr antibody to document protein phosphorylation. α-GFP antibody was used to show expression level of GFP-PhpP and immunodetection of RpoA was used as a loading control. Position of StkP and its substrates is indicated by arrows. **b** Morphology of strains expressing GFP-PhpP-WT (Sp140), GFP-PhpP-D192A (Sp292) and GFP-PhpP-D231A (Sp293) in Δ*phpP* genetic background. Pneumococcal strains were cultivated in C + Y medium supplemented with ZnSO_4_. Phase contrast images show cell morphology in the presence of 0.2 and 0.3 mM ZnSO_4_ and median cell lengths ± MAD (*n* = 300) corresponding to each image are shown below. Bar, 5 μm. **c** Cell length analysis. Cell length parameters were analyzed and plotted in box-and-whiskers graph. Mann-Whitney *U* test: * cell length in the presence of inducer is significantly different from uninduced conditions (0 mM ZnSO_4_) *P* < 0.0001. 300 cells were scored per sample. **d** Localization of PhpP. Strains expressing GFP-PhpP-WT (Sp140), GFP-PhpP-D192A (Sp292) and GFP-PhpP-D231A (Sp293) were cultivated in C + Y medium supplemented with 0.2 mM ZnSO_4_. GFP signal and overlay of phase contrast and GFP signal are shown. Enrichment of PhpP at midcell of cells at first stage of cell division (predivisional cells) is indicated by full arrow; cells showing cytoplasmic localization of PhpP are indicated by open arrow. Bar, 1 μm. Predivisional cells (*n* = 20) showing either midcell enrichment of PhpP (WT and D192A) or cytoplasmic localization (D231A) were selected to quantify distribution of GFP-PhpP along the cell axis. Fluorescence intensity (arbitrary units) versus cell length is plotted in corresponding graphs (error bars show SD)

PhpP contains two conserved aspartate residues, D192 and D231, which are directly involved in metal ions binding and are essential for the activity of eukaryotic PP2C phosphatases [37]. Previously, we reported that substitution of D192 and D231 for alanine abolished PhpP activity in vitro [17]. Here, we investigated the importance of D192 and D231 for enzymatic activity and localization of PhpP in vivo. We constructed strains expressing PhpP mutant alleles D192A and D231A fused to GFP under an inducible P_Zn_ promoter in the Δ*phpP* genetic background. Strains expressing GFP-PhpP-WT (Sp140), the D192A allele (Sp292) and the D231A allele (Sp293) were cultivated in C + Y medium with or without zinc, and PhpP expression and the phosphorylation pattern were detected using specific antibodies. Expression of PhpP-WT and PhpP-D192A was similar; however, the expression of PhpP-D231A was lower as indicated by immunodetection with the α-GFP antibody (Fig. 5a). Immunodetection with the α-pThr antibody showed that increasing the expression of the wild type allele resulted in a decrease in overall phosphorylation intensity. On the other hand, increased expression of PhpP-D192A or D231A upon addition of zinc did not affect the phosphorylation intensity in strains Sp292 and Sp293, respectively, indicating that both alleles are catalytically inactive. Phase contrast microscopy revealed that the morphology of strain Sp140 expressing GFP-PhpP-WT changed depending on zinc concentration, and cell length increased (Fig. 5b). On the other hand, the morphology of strains expressing the mutant alleles of PhpP did not change (Fig. 5b) indicating inability to complement the mutant phenotype. Cell size analysis confirmed these observations (Fig. 5c). PhpP-WT expression in the Sp140 strain led to increases in cell length up to 2.11 ± 0.36 μm when 0.3 mM ZnSO_4_ was added to the medium, while cell length remained unchanged in strains Sp292 (PhpP-D192A) (1.49 ± 0.2 μm) and Sp293 (PhpP-D231A) (1.43 ± 0.23 μm).

Previously, we demonstrated that the protein phosphatase PhpP is localized in the cytoplasm, but it is significantly enriched at the midcell during the early exponential phase of growth, and this localization depends on the presence of active StkP [20]. To determine localization of catalytically inactive GFP-PhpP, we cultivated strains Sp140, Sp292 and Sp293 in medium supplemented with 0.2 mM ZnSO_4_ until the early exponential phase (OD_600_ 0.2) and examined live cells using fluorescence microscopy. PhpP-WT was clearly associated with the cell division septum in 23 % (176/766) of the cells and showed cytoplasmic distribution in 77 % of cells (Fig. 5d). PhpP-D192A was enriched at the midcell in 19 % (152/800) of the cells; however, the GFP signal in these cells was more diffuse (Fig. 5d). To quantify the difference, we measured the fluorescence intensity profiles in the cells at the first stage of cell division (predivisional cells). We confirmed different distributions of the GFP-PhpP-WT and GFP-PhpP-D192A signals along the cell axis, which indicates that PhpP-D192A is more abundant in the cytoplasm (Fig. 5d). Interestingly, PhpP-D231A was localized exclusively in the cytoplasm (Fig. 5d). These data suggest that mutant alleles of PhpP not only loose the catalytic activity but also lose the ability to co-localize with cell division apparatus.

### Jag protein (Spr1851) is a previously unknown substrate of StkP and PhpP

**Fig. 6:**
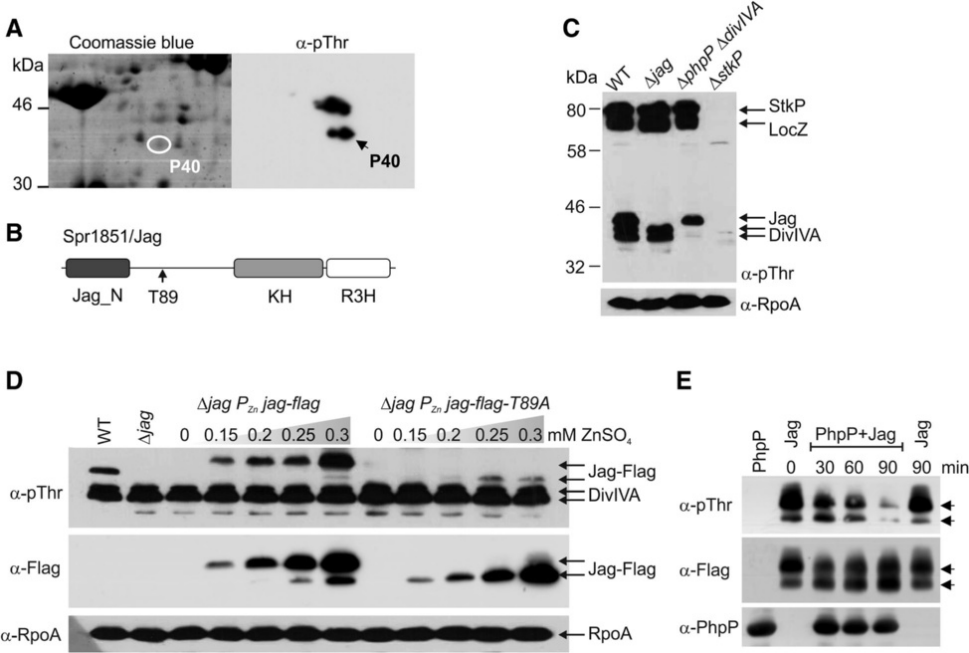
Spr1851/Jag is a new substrate of StkP and PhpP. **a** Identification of Jag. Membrane fraction isolated and extracted by TFE/chloroform from strain Sp169 (Δ*phpP* Δ*divIVA*) was resolved on 2D SDS-PAGE, immunobloted with α-pThr antibody and matched with parallel gel stained with Coomassie Blue. Spot corresponding to P40 was excised, analyzed by MALDI-MS and identified as Spr1851. **b** Schematic structure of Spr1851/Jag. Jag_N: conserved domain found at the N-terminus of Jag proteins; T89: phosphorylated threonine 89; KH: single-stranded RNA binding domain; R3H: putative single-stranded nucleic acid binding domain. **c** Deletion of *jag*. Whole cell lysates of WT (Sp1), Δ*jag* (Sp295), Δ*phpP* Δ*divIVA* (Sp169) and Δ*stkP* (Sp10) were separated by SDS-PAGE and immunoblotted with α-pThr antibody to detect protein phosphorylation. Immunodetection of α-subunit of RNA polymerase (α-RpoA) was used as a loading control. Arrows indicate position of StkP and its substrates. **d** Phosphorylation of T89. Total protein lysates (30 μg) of WT (Sp1), Δ*jag* (Sp295), complementation strain Δ*jag* P_Zn_-*jag-flag* (Sp304) and Δ*jag* P_Zn_-*jag-flag-T89A* (Sp302) were separated by SDS-PAGE and immunoblotted with α-pThr antibody to detect protein phosphorylation. Expression of Jag-Flag was monitored by immunodetection with anti-Flag antibody (α-Flag) and immunodetection of RpoA (α-RpoA) was used as a loading control. Arrows indicate position of proteins. **e** Dephosphorylation of Jag. Purified Jag-Flag was incubated with His-PhpP in vitro and reaction was stopped at given time. Samples were subjected to SDS-PAGE and immunoblotted with α-pThr antibody to visualize dephosphorylation in time. PhpP-His was detected with α-PhpP antibody and Jag-Flag was detected with α-Flag antibody. To exclude the spontaneous decay, phosphorylated form of Jag-Flag was incubated for 90 min in phosphatase reaction buffer without addition of His-PhpP. Arrows indicate position of two differentially phosphorylated forms of Jag

The elevated level of Thr phosphorylation in the *phpP* null mutant helped us to detect phosphorylated membrane proteins previously unrecognized in wild type lysates. Because the phosphorylated proteins designated P35 and P40 migrate close to phosphorylated DivIVA, we generated strain Δ*phpP*Δ*divIVA* (designated Sp169), which would enable appropriate separation of the new substrates. To identify these StkP substrates we extracted proteins from the membrane fraction using trifluorethanol (TFE) [38] and separated them using two-dimensional (2D) SDS-PAGE as described in the *Methods*. The protein spot corresponding to P40 was successfully resolved, and its phosphorylation was confirmed by immunoblotting (Fig. 6a). The protein spot was excised, digested by trypsin and identified using MALDI-TOF mass spectrometry as Spr1851, a homolog of Jag/SpoIIIJ-associated protein from *B. subtilis*. We named the product of the *spr1851* gene Jag_Spn_ (Fig. 6b). Jag_Spn_ contains an N-terminal Jag_N domain and KH domain followed by an R3H domain at the C-terminus (Conserved Domain Database (CDD) [39]) (Fig. 6b). The Jag_N domain located at the N-terminus of bacterial Jag proteins is a conserved stretch of 50 amino acids without a defined function (CDD). The KH domain is a single-stranded nucleic acid-binding domain that mediates RNA target recognition in proteins that regulate gene expression in eukaryotes and prokaryotes (reviewed in [40]). The R3H motif is present in proteins from a diverse range of organisms that includes Eubacteria, green plants, fungi and various groups of metazoans, and it is predicted to bind ssDNA or ssRNA in a sequence-specific manner. Jag homologues are conserved in bacteria, especially in *Firmicutes*, and their domain architecture suggests that they bind RNA; however, their function is unknown.

To verify the phosphorylation of Jag_Spn_ we constructed a *jag* null mutant named Sp295 using the Janus cassette strategy described in the *Methods* *.* We detected phosphorylated proteins in whole cell lysates of the wild type, Δ*jag* and Δ*stkP* strains and compared them with strain Δ*phpP*Δ*divIVA* to better distinguish different phosphoprotein bands. Immunoblotting showed that a phosphoprotein corresponding to 40 kDa (P40) is present in the wild type and Δ*phpP*Δ*divIVA* strain but absent in the Δ*jag* strain, which indicates that Spr1851/Jag corresponds to StkP substrate P40 (Fig. 6c).

To verify further phosphorylation of Jag we prepared the complementation strain Sp304 expressing Jag with a Flag-tag at the C-terminus (Jag-Flag) under the inducible P_Zn_ promoter in the Δ*jag* genetic background. Figure 6d shows that addition of zinc induced expression of Jag-Flag in strain Sp304 (Δ*jag* P_Zn_-*jag-flag*), and immunodetection with the α-pThr antibody confirmed that the protein is phosphorylated. A second, faster migrating band of Jag was detected with the α-Flag antibody when ZnSO_4_ was added at concentrations of 0.25 and 0.3 mM. This protein band interacted weakly with the α-pThr antibody, which indicated that this form of Jag is also phosphorylated.

### Jag is phosphorylated on Thr89

Spr1851/Jag was previously found to be phosphorylated on Thr89 in a global study published by Sun et al. [41]. Thr89 is located in a region that does not show significant homology to any conserved domain; however, Thr89 is conserved in many streptococcal species (KEGG, Kyoto Encyclopedia of Genes and Genomes). To verify phosphorylation of Thr89, we mutagenized this residue to unphosphorylatable alanine and constructed strain Sp302 expressing the phosphoablative allele *jag-flag-T89A* under the P_Zn_ promoter in the Δ*jag* genetic background*.* Upon induction of expression, we detected production of Jag-Flag-T89A migrating faster than the major form of Jag-Flag-WT. This suggests that alteration of mobility is related to the unphosphorylated state (Fig. 6d). Immunodetection with the α-pThr antibody showed a significant decrease in phosphorylation; however, the signal was not completely lost, and Jag-T89A reacted weakly with the α-pThr antibody when expressed at higher levels (0.25–0.3 mM ZnSO_4_) (Fig. 6d, lanes 11, 12). These results confirm that Thr89 is indeed a phosphoacceptor residue in vivo; however, another still unidentified secondary phosphorylation site is present in Jag.

### Jag is dephosphorylated by PhpP

To demonstrate that PhpP directly dephosphorylates Jag, we isolated phosphorylated Jag-Flag from cell lysates of strain Sp304 via affinity chromatography and performed in vitro dephosphorylation reactions as described in the *Methods*. Phosphorylation of Jag was monitored by immunodetection with the α-pThr antibody, and the results showed that the loss of the phosphorylation signal was time dependent (Fig. 6e). This experiment confirmed that PhpP directly dephosphorylates Jag. Two different forms of Jag were detected upon incubation with PhpP, correlating with our finding that Jag most likely contains more than one phosphorylated residue (Fig. 6d).

### Characterization of the Δ*jag* phenotype

**Fig. 7:**
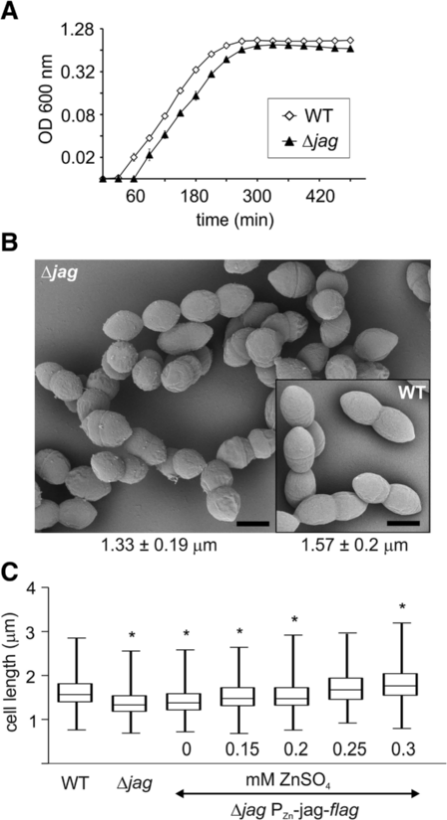
Phenotype of Δ*jag* mutant. **a** Growth curve of WT (Sp1) and Δ*jag* (Sp295) cultivated in TSB medium plotted in semi logarithmic scale. Data shown represent mean ± SD for three independent experiments. Where error bars are not shown, the SD was within the size of the symbol. **b** Scanning electron microscopy of WT (Sp1) and Δ*jag* strain (Sp295). Bar 0.5 μm. Cell length is expressed as median value ± MAD (*n* = 300). **c** Cell size analysis of WT (Sp1), Δ*jag* (Sp295) and complementation strain Δ*jag* P_Zn_-*jag-flag* (Sp304). Cell length parameters measured with MicrobeTracker software were analyzed and plotted in box-and-whiskers graph. Mann-Whitney *U* test: * cell length of mutant strain is significantly different from that of the WT strain *P* < 0.0001. 300 cells were scored per sample

To obtain insight into Jag function in pneumococcus, we characterized the phenotype of a Δ*jag* mutant. The Δ*jag* mutant showed retarded growth in TSB medium, with a longer doubling time (32 min) than the wild type (29 min). The mutant had a significantly longer lag phase and reached stationary phase at a lower optical density (Fig. 7a). Phase contrast microscopy indicated that mutant cells are smaller, a phenotype reminiscent of the Δ*phpP* mutant. Cell size analysis confirmed that the median cell length (1.33 ± 0.19 μm) and cell width (0.59 ± 0.01 μm) of the mutant were significantly smaller than the median cell length and width of the wild type (1.57 ± 0.2 μm and 0.67 ± 0.09 μm) (*P* < 0.0001; Mann-Whitney rank sum test). Scanning electron microscopy further supported these data; however, no significant abnormalities in cell shape and morphology were observed (Fig. 7b). We also determined cell size of the complementation strain Sp304 (Δ*jag* P_Zn_-*jag-flag*) upon addition of increasing zinc concentrations. Induced expression of Jag-WT resulted in complementation characterized by increasing cell length (Fig. 7c). This rescue of the phenotype confirmed the relationship between inactivation of *jag* and decreased cell dimensions. The cells reached wild type cell length at a concentration of approximately 0.25 mM ZnSO_4_ (1.67 ± 0.25 μm). The cell length increased further upon addition of 0.3 mM ZnSO_4_ until it reached 1.76 ± 0.26 μm, suggesting that overexpression of Jag led to significant cell elongation. These data suggest that Jag plays a role in pneumococcal cell division and helps to maintain proper cell shape.

The *jag* homologue in *B. subtilis* forms a bicistronic operon with the *spoIIIJ* gene [31], which corresponds to pneumococcal *spr1852* encoding the YidC1/Oxa1 membrane protein insertase. This gene cluster is widely conserved (KEGG). YidC homologues are required for the insertion and/or proper folding of integral membrane proteins (reviewed in [42]). Most Gram-positive bacteria encode two YidC paralogues, YidC1 and YidC2, which correspond to Spr1852 and Spr1790, respectively, in *S. pneumoniae*. The role of YidC homologues in *S. pneumoniae* has not been described; however, in *S. mutans*, disruption of YidC2 results in a loss of genetic competence, decreased membrane-associated ATPase activity and stress sensitivity. Loss of YidC1 has less severe defects, with little observable effect on growth or stress sensitivity [43]. Although the two insertases have different physiological functions, both of them contribute to biofilm formation and cariogenicity in rats [43].

The Jag association with the membrane and likely co-transcription with *yidC1* suggest that both proteins might be functionally linked. It is tempting to speculate that Jag_Spn_ plays an indirect role in targeting of the integral membrane proteins. Given the Δ*jag* phenotype and its phosphorylation by StkP, which regulates cell division in pneumococcus, Jag_Spn_ might specifically affect targeting of cell division proteins. YidC homologues are involved in cell division processes in different bacteria. The well-studied YidC1/SpoIIIJ in *B. subtilis* is required for sporulation [31]. Interestingly, the cell division proteins FtsQ and FtsEX have been found to be substrates of YidC in *E. coli* and *Shigella*, respectively [44, 45]. A recent report also showed that YidC assists in the biogenesis of penicillin-binding proteins (PBP) in *E. coli*, and in the absence of YidC, two critical PBPs, PBP2 and PBP3, are not correctly folded, and their substrate-binding capacity is reduced, although the total amount of protein in the membrane is not affected [46].

## Conclusions

*Streptococcus pneumoniae* has a characteristic ovoid shape, which is most likely achieved by the concerted action of two peptidoglycan biosynthetic machineries: peripheral and septal [47, 48]. We previously proposed a model in which Ser/Thr protein kinase StkP coordinates cell wall synthesis and cell division in *S. pneumoniae* [20]. Here, we demonstrate that the cognate Ser/Thr protein phosphatase PhpP is not essential as published previously [10, 21, 28] and plays an opposing role in cell division to that of StkP. Overexpression of PhpP, which leads to dephosphorylation of StkP substrates, mimics the *stkP* null phenotype and the dividing cells are elongated and contain multiple unconstricted cell division septa. In the absence of *phpP* we observe enhanced autophosphorylation of StkP and hyperphosphorylation of StkP substrates. We show that PhpP regulates not only activity of StkP but dephosphorylates directly StkP substrates. The morphology of Δ*phpP* cells resembles StkP overexpression, and the cells do not achieve the size of the wild type, most likely due to insufficient elongation of cells or premature constriction of the Z-ring. We hypothesize that PhpP and StkP co-ordinately regulate the shift from peripheral to septal cell wall synthesis through phosphorylation of several substrates, including cell division proteins. In contrast, we did not confirm a straightforward regulatory impact of PhpP on the other functions of StkP. Characterization of the *phpP* null mutant revealed that like the *stkP* null mutant, it is more sensitive to elevated temperature, oxidative stress and that both mutant strains have reduced competence for genetic transformation. These results suggest that unbalanced activity of each of these enzymes is critical for bacterial physiology. We cannot exclude the possibility that PhpP may also have broader substrate specificity and may dephosphorylate phosphoproteins other than StkP substrates. Detection of proteins specifically phosphorylated in the Δ*phpP* strain allowed us to identify new substrate modified by StkP/PhpP couple. The product of gene *spr1851* called Jag_Spn_ is a putative RNA binding protein phosphorylated on Thr89. Jag proteins are widely conserved in bacteria and their role is unknown. Phenotype of the Δ*jag* mutant suggests that Jag_Spn_ is involved in cell division and maintaining proper cell shape of *S. pneumoniae*.

## Methods

### Bacterial strains and growth conditions

The bacterial strains used in this study are listed in Table 1. *E. coli* DH5α used as a general purpose cloning host and *E. coli* BL21 used for protein expression were cultured in Luria–Bertani (LB) broth at 37 °C. The wild type *S. pneumoniae* strain Rx1 and its corresponding mutants were grown statically at 37 °C in Brain-heart Infusion (BHI) medium, Tryptone Soya Broth (TSB) medium, semi-synthetic C medium supplemented with 0.1 % yeast extract (C + Y) [49] or in Casein Tryptone (CAT) medium supplemented with 0.2 % glucose and 1/30 volume 0.5 M K_2_HPO_4_, pH 7.5 [49]. DNA from the strain CP1016 (rif-23) was used as the donor DNA for the competence assays [50]. The following antibiotics were added when necessary at the indicated concentrations (in μg ml^−1^): rifampin (Rif), 1; kanamycin (Kan), 200; streptomycin (Sm), 500; tetracycline (Tet), 2.5; erythromycin (Erm), 1 (for *S. pneumoniae*); ampicillin (Amp), 100; kanamycin (Kan), 50; erythromycin (Erm), 100 (for *E. coli*).

**Table 1:** Bacterial strains and plasmids used in this study

Strain/plasmid	Genotype or description	Source
Strains		
*S. pneumoniae*		
Sp1 (Rx1)	unencapsulated wild-type, *str1*; *hexA*	[54]
Sp10	*Cm*, *stkP*::*cm*	[17]
Sp26	*Cm*, *divIVA*::*cm*	[55]
Sp57	*locZ::lox72*	[25]
Sp100	*Kan*, *phpP*::*kan rpsL*	This work
Sp113	Δ*phpP*	This work
Sp120	*Tet*, Δ*phpP bga*::P_Zn_-*phpP*	This work
Sp140	*Tet*, Δ*phpP bga*::P_Zn_-*gfp*-*phpP*	This work
Sp161	*Kan*, Δ*phpP divIVA::kan rpsL*	This work
Sp169	Δ*phpP* Δ*divIVA*	This work
Sp174	*Erm*, Δ*divIVA pMU-P96-divIVA-flag*	This work
Sp188	*Erm*, Δ*locZ pMU-P96-flag-locZ*	This work
Sp220	*Kan, phpP*::*kan rpsL* (reverted from Sp113)	This work
Sp222	wild-type (reverted from Sp113)	This work
Sp292	*Tet*, Δ*phpP bga::*P_Zn_ *-gfp-phpP-D192A*	This work
Sp293	*Tet*, Δ*phpP bga::*P_Zn_ *-gfp-phpP-D231A*	This work
Sp295	Δ*jag*	This work
Sp302	*Tet*, Δ*jag bga::*P_Zn_ *-jag-flag-T89A*	This work
Sp304	*Tet*, Δ*jag bga::*P_Zn_ *-jag-flag*	This work
*E. coli*		
DH5α	*F- Φ80lacZΔM15 Δ(lacZYA-argF) U169 recA1 endA1 hsdR17 (rk-, mk+) phoA supE44 λ- thi-1 gyrA96 relA1*	Invitrogen
BL21	*F- ompT gal [dcm][lon] hsdSB (rB- mB-) (DE3)*	Novagen
Plasmids		
pJWV25	*Amp, tet, bgaA*, P_Zn_-*gfp*+	[35]
pZn-PhpP	*Amp, tet, bgaA*, P_Zn_-*phpP*	This work
pZn-flag-locZ	*Amp, tet, bgaA,* P_Zn_ *-flag-locZ*	This work
pJWV25-phpP	*Amp, tet, bgaA*, P_Zn_-*gfp-phpP*	[20]
pZn-gfp-phpP-D192A	*Amp, tet, bgaA,* P_Zn_ *-gfp-phpP-D192A*	This work
pZn-gfp-phpP-D231A	*Amp, tet, bgaA,* P_Zn_ *-gfp-phpP-D231A*	This work
pZn-jag-flag-T89A	*Amp, tet, bgaA,* P_Zn_ *-jag-flag-T89A*	This work
pZn-jag-flag	*Amp, tet, bgaA,* P_Zn_ *-jag-flag*	This work
pEXphpP-D231A	*Kan*, *phpP-D231A*	[17]
pMU1328	*Erm,* empty vector	[51]
pMU-P96-divIVA-flag	*Erm,* P*96-divIVA-flag*	This work
pMU-P96-flag-locZ	*Erm,* P*96-flag-locZ*	This work
Janus cassette	*Kan, kan-rpsL* ^*+*^	[52]

*Amp* ampicillin resistance marker, *cm* chloramphenicol resistance marker, *kan* kanamycin resistance marker, *tet* tetracycline resistance marker, *erm* erythromycin resistance marker

### Plasmid construction

Plasmids used in this study are listed in Table 1 and oligonucleotides are listed in Additional file 1: Table S1. To construct plasmid pZn-PhpP, *phpP* was amplified with primers JG19 and JG20 using WT chromosomal DNA as a template. The P_*czcD*_ (P_Zn_) promoter was amplified with primers LN123 and JG21 from a template plasmid pJWV25 [35]. Both PCR products were used as a template in a fusion PCR with primers LN123 and JG20. The final PCR product P_Zn_-*phpP* was cloned into the KpnI and NotI restriction sites of the plasmid pJVW25. Plasmid pZn-jag-flag was constructed as follows: *jag* gene was amplified with primer pairs AU77 and AU79 (containing Flag sequence and NotI restriction site) using wild type chromosomal DNA as a template. The P_Zn_ promoter was amplified with primers LN123 and AU76 from a template plasmid pJWV25. Both PCR products were used as a template in a fusion PCR with primers LN123 and AU79. The final PCR product was cloned into the EcoRI and NotI restriction sites of the plasmid pJVW25. To generate pZn-flag-locZ, the P_Zn_ promoter was amplified with primers LN123 and NS1 from a template pJWV25 and *locZ* was amplified with primers NS2 (containing Flag sequence) and LN155 using wild type chromosomal DNA as a template. Both PCR products were used as a template in a fusion PCR with primers LN123 and LN155 and the final PCR product was digested and cloned into the EcoRI and NotI restriction sites of the plasmid pJVW25.

To construct plasmid pMU-P96-flag-locZ, the P96 promoter was amplified with primer LN231 and LN215 from a template plasmid pMU1328 [51]. The *flag-locZ* fragment was amplified with primers NS3 and NS4 using pZn-flag-locZ as a template. Both PCR products were used as a template in a fusion PCR with primers LN231 and NS4. The resulting PCR fragment was cloned into EcoRI and SalI sites of pMU1328 vector. To generate plasmid pMU-P96-divIVA-flag, the P96 promoter was amplified with primers LN214 and LN215 from a template pMU1328. The *divIVA* gene was amplified with primers LN218 and LN229 (containing Flag sequence) using wild type chromosomal DNA as a template. Both PCR fragments were fused in a fusion PCR with primer pair LN214/LN229 and the acquired PCR fragment was inserted into BamHI and SalI sites of pMU1328. All constructs were verified by DNA sequencing.

### Site directed mutagenesis

To introduce specific mutations in the *phpP* and *jag* genes we used the QuickChange mutagenesis kit (Stratagene) according to manufacturer’s instructions. T89A mutation was introduced into plasmid pZn-jag-flag using primer pair AU80/AU81 to generate plasmid pZn-jag-flag-T89A. D192A mutation was introduced into pJWV25-phpP plasmid using primer pair AU69/AU70 to generate plasmid pZn-gfp-phpP-D192A. Plasmid pZn-gfp-phpP-D231A was constructed as follows: gene *phpP-D231A* was amplified by PCR using plasmid pEXphpP-D231A as a template and primers AU67 and AU68 containing SpeI and NotI restriction site, respectively. PCR product was cloned into pJWV25 generating pZn-gfp-phpP-D231A. All constructs were verified by DNA sequencing.

### Construction of pneumococcal strains

pJWV25 derived strains expressing proteins under control of P_Zn_ promoter were prepared by transformation of *S. pneumoniae* competent cells with the corresponding pJWV25 derived plasmids previously linearized by digestion with PvuI. Tetracycline resistant transformants were obtained by a double-crossover recombination event between the chromosomal *bgaA* gene of the parental strain and *bgaA* regions located on the plasmids as described previously [35]. Following plasmids were used for construction of corresponding strains: pZn-PhpP: Sp120; pJWV25-phpP: Sp140; pZn-gfp-phpP-D192A: Sp292; pZn-gfp-phpP-D231A: Sp293; pZn-jag-flag: Sp304; pZn-jag-flag-T89A: Sp302. Strain Sp174 was prepared by transformation of strain Sp26 (Δ*divIVA*) with plasmid pMU-P96-divIVA-flag. Strain Sp188 was prepared by transformation of strain Sp57 (Δ*locZ*) with pMU-P96-flag-locZ.

Strain Sp113 (Δ*phpP*) was constructed as described by Agarwal et al*.* [11], using a Janus cassette (kanamycin resistance gene followed by the recessive *rpsL* gene)-based two-step negative selection strategy [52]. In the first step 1100 bp and 1037 bp fragments corresponding to the upstream and downstream flanking regions of the *phpP* gene were amplified from the wild type chromosomal DNA with JG24/JG25 and JG26/JG27 primer pairs, respectively. The Janus cassette (1333 bp) amplified by JG28/JG29 primers from the Janus casette DNA fragment was attached to the *phpP* flanking regions by fusion PCR using primers JG24 and JG27. The resulting PCR fragment was used for the transformation of the *S. pneumoniae* strain Rx1, and Kan^R^/Sm^S^ transformants (Sp100, *phpP*::*kan rpsL*) were selected. The PCR fragments, consisting of the upstream and downstream flanking region of the *phpP* gene, were amplified by JG24/JG31 and JG27/JG30 primer pairs, respectively, and fused by overlap extension using primers JG24/JG27. The resulting fragment was transformed into the strain Sp100 to gain Sp113 (Sm^R^/Kan^S^). Reverted strain Sp222 (WT_R_) was constructed as follows: in the first step, Δ*phpP* strain (Sp113) was transformed by PCR fragment consisting of upstream and downstream flanking regions of the *phpP* gene fused with Janus cassette, as described in the previous section and Sm^S^/Kan^R^ transformants (strain Sp220, *phpP*::*kan rpsL*) were selected. The PCR fragments, consisting of the upstream and downstream flanking region of the *phpP* gene, were amplified by JG24/JG68 and JG27/JG67 primer pairs, respectively, and fused by overlap extension with *phpP* gene (amplified by primers JG65 and JG66) using the primers JG24/JG27. The resulting fragment was transformed into the strain Sp220 to obtain reverted strain Sp222 (Sm^R^/Kan^S^).

Strain Sp295 (Δ*jag*) was constructed using a Janus cassette strategy [52]. In the first step, upstream and downstream flanking regions of the *jag* gene were amplified from the wild type chromosomal DNA with AU57/AU58 and AU59/AU60 primer pairs, respectively. The Janus cassette (1333 bp) amplified by JG28/JG29 primers from the Janus casette DNA fragment was attached to the *jag* gene flanking regions by fusion PCR using primers AU57 and AU60. The resulting PCR fragment was used for the transformation of the *S. pneumoniae* strain Rx1, and Sm^S^/Kan^R^ transformants (*jag*::*kan rpsL*) were selected. The PCR fragments, consisting of the upstream and downstream flanking region of the *jag* gene, were amplified by AU57/AU74 and AU60/AU75 primer pairs, respectively, and fused by overlap extension using the primers AU57/AU60. The resulting fragment was transformed into the *jag::kan rpsL* strain and Sm^R^/Kan^S^ transformants were selected (strain Sp295).

Strain Sp169 (Δ*phpP* Δ*divIVA*) was constructed as follows: the upstream and downstream flanking regions of the *divIVA* gene were amplified from the wild type chromosomal DNA with JG57/JG58 and JG59/JG60 primer pairs, respectively. Both flanking regions were attached to the amplified Janus cassette (see above) by fusion PCR using primers JG57 and JG60. The resulting PCR fragment was used for the transformation of the Δ*phpP* strain (Sp113), and Sm^S^/Kan^R^ transformants were selected to obtain Sp161 (Δ*phpP divIVA*::*kan rpsL*). To construct deletion of *divIVA* gene without selectable marker, the PCR fragments, consisting of the upstream and downstream flanking region of the *divIVA* gene, were amplified by JG57/JG62 and JG60/JG61 primer pairs, respectively, and fused by overlap extension using the primers JG57/JG60. Resulting fragment was transformed into Sp161 strain to yield Sm^R^/Kan^S^ strain named Sp169.

### Western blot analysis and immunodetection

Cells were grown in C + Y medium with or without the addition of an appropriate concentration of ZnSO_4_ to an OD_600_ 0.4, harvested and resuspended in 1 ml of precooled lysis buffer containing 25 mM Tris (pH 7.5), 100 mM NaCl, Benzonase (Merck), and protease inhibitors (Roche). Cells were disintegrated using glass beads in a FastPrep homogenizer (ThermoScientific). Cell debris was pelleted by a centrifugation at 5 000 × g. The total cell lysate was further fractionated by centrifugation at 100 000 × g for 1 h at 4 °C and the cytoplasmic and membrane fractions were obtained. The protein concentration was determined using a bicinchoninic acid (BCA) protein estimation kit (Pierce). An aliquot of 30 μg of each protein fractions or total cell lysate was diluted in 1 × SDS loading buffer and boiled for 10 min. After SDS-PAGE separation, proteins were transferred to a PVDF membrane by Western blotting. Phosphorylated proteins were detected using anti-phosphothreonine polyclonal rabbit antibody (Cell Signalling, 9381S, LOT 22). StkP, PhpP, LocZ and RpoA were detected using specific custom made polyclonal rabbit sera derived from rabbits immunized with corresponding purified full length His-tagged proteins (Apronex, Czech Republic) and were used as described previously [20, 23, 25]. Specificity of anti-StkP and anti-PhpP antibody is documented by loss of reactivity in corresponding mutant strains (Fig. 1b; [20]). Specificity of anti-LocZ antibody is documented by loss of reactivity in Δ*locZ* strain [25]. Specificity of antibody against house-keeping gene product RpoA is documented by reactivity with pure protein [23]. Flag-tagged and GFP-tagged proteins were detected with anti-Flag rabbit (Sigma-Aldrich, F7425, LOT 064M4757V) and anti-GFP mouse (Santa Cruz Biotech, sc-9996, LOT A1111) antibody. Protein abundance was measured using ECL detection substrate (Pierce) and signal was developed using G:Box Chemi XRQ instrument (SynGene) or by exposition on medical X-ray film (Agfa). To quantify protein phosphorylation in cell lysates the immunoblot was scanned by G:Box Chemi XRQ instrument to obtain linear range of exposure and signal was analyzed by Quantity One software, version 4.6.3 (Bio-Rad). Data represent mean ± standard deviation (SD) from three independent experiments and were normalized to the total protein level.

### Trifluoroethanol/chloroform extraction, two-dimensional (2D) SDS-PAGE and mass spectrometry

Protein membrane fractions were extracted by trifluoroethanol/chloroform mixture as described previously [38]. Resulting aqueous and insoluble fractions were solubilized in lysis buffer (7 M urea, 2 M thiourea, 2 % Triton X-100, 0,5 % amido sulfobetaine-14 (ASB14), 1 % Ampholytes 3–10, 50 mM DTT) and purified with 2-D Clean-Up Kit (GE Healthcare). The protein concentration was determined using 2-D Quant kit (GE Healthcare). 2D SDS-PAGE was performed on IPG strips pH 4–7 NL (Amersham Biosciences) as described previously [23]. Gels were either stained with colloidal Coomassie Brilliant Blue G-250 (CBB G-250) or electroblotted. The protein spots selected for mass spectrometric analysis were destained using 50 mM 4-ethylmorpholine acetate (pH 8.1) in 50 % acetonitrile (MeCN) and in-gel digested overnight with trypsin (100 ng; Promega) in a cleavage buffer containing 25 mM 4-ethylmorpholine acetate. The resulting peptides were extracted to 40 % MeCN/0.1 % TFA and measured on an Ultraflex III MALDI-TOF mass spectrometer (BrukerDaltonics) in a mass range of 700–4000 Da. For protein identification the peptide mass spectra were searched against SwissProt or NCBI (National Center for Biotechnology Information) bacterial database using an in-house Mascot search engine. The identity of protein candidates was confirmed using MS/MS analysis.

### Competence assays

*S. pneumoniae* was induced to competence using competence stimulating peptide (CSP) as described previously [36], with minor modifications. Briefly, an exponential culture of cells cultivated in BHI medium was diluted 1:20 in BHI supplemented with 0.2 % BSA (Bovine Serum Albumin) and 1 mM CaCl_2_ and pH adjusted to 7.8. The recipient strain was activated by the addition of CSP (250 ng ml^−1^) and incubated for 10 min at room temperature. The *rif-23* donor DNA (1 μg ml^−1^) was then added and DNA uptake was obtained by 20 min incubation at room temperature. The mixture was then diluted 1:10 in BHI medium and incubated at 37 °C for 2 h. Serial dilutions of transformed cultures were plated, and transformation efficiencies were calculated as the ratio of the viable counts on plates with and without rifampin. To generate natural competence profiles of the wild type and mutant strains, method according to Echenique et al. [36] was used with several modifications. Briefly, stocks of bacteria grown in BHI medium to an OD_600_ of 0.5 were diluted 100-fold in the same medium supplemented with 0.2 % BSA and 1 mM CaCl_2_ and pH adjusted to 7.8 and grown at 37 °C. Samples were withdrawn at 15-min intervals, diluted 10-fold into BHI medium containing *rif-23* donor DNA, and incubated for 30 min at 30 °C. Further incubation was carried out at 37 °C for 90 min before plating serial dilutions with and without rifampin. Transformation efficiencies were calculated as the ratio of the viable counts on plates with and without rifampin.

### Growth and environmental stress tolerance

To generate the growths curves pneumococcal strains were inoculated (6.8 × 10^5^ CFU ml^−1^) in TSB medium, cultivated statically and the growth was monitored every 30 min by measuring OD_600_ for period of 7 to 8 h. The tolerance of pneumococcal strains to environmental stress was examined in a manner similar to that described previously [19]. To investigate heat stress resistance cultures were inoculated into TSB medium prewarmed to 37 and 40 °C. The acid tolerance of all strains was monitored by measuring the growth curve in TSB medium adjusted to pHs 6.5 and 7.5. The alkaline tolerance was monitored at pH 8.0. To test the tolerance to osmotic stress, bacteria were first grown to early exponential phase (OD_600_ 0.2) and then inoculated into prewarmed TSB medium with or without 400 mM NaCl. The sensitivity of cells to H_2_O_2_ was tested by exposing exponential cultures (OD_600_ 0.4) grown in CAT medium at 37 °C to 10 mM and 20 mM H_2_O_2_ for 15 min. Viable cell counts were determined by plating serial dilutions of cultures onto agar plates before and after exposure to H_2_O_2_. The results were expressed as percentages of survival.

### Protein purification and dephosphorylation assay

Recombinant His-PhpP was purified as described previously [17]. To purify Flag-tagged proteins form *S. pneumoniae* the strains Sp174 (Δ*divIVA pMU-P96-divIVA-flag*), Sp188 (Δ*locZ pMU-P96-flag-locZ*) and Sp304 (Δ*jag bga::* P_Zn_ *-jag-flag*) were grown statically at 37 °C in C + Y medium supplemented with 0.25 mM ZnSO_4_. Total cell lysates were prepared as described above and Flag-tagged proteins were purified by affinity chromatography using ANTI-Flag M2 Affinity Gel (Sigma-Aldrich) according to the manufacturer’s instructions. Dephosphorylation assay was performed basically as described previously [17]. Briefly, Flag fusion-proteins of interest (Flag-LocZ, DivIVA-Flag or Jag-Flag) bounded on the M2 affinity gel were mixed with 55 μl of reaction buffer and 4 μg of purified His-PhpP and incubated at 37 °C. Phosphatase reaction was terminated by the addition of 5× SDS-PAGE sample buffer at different time intervals (0–90 min). Samples were boiled, subjected to SDS-PAGE and immunoblotted as described above.

### Electron microscopy

Samples for electron microscopy were prepared as described elsewhere [25] except the dried samples were sputter coated with 3 nm of platinum in a Q150T ES sputter coater (Quorum Technologies Ltd.). The final samples were examined in a FEI Nova NanoSem 450 scanning electron microscope (FEI Czech Republic s.r.o.) at 5 kV using Circular Backscatter Detector and back-scattered electrons.

### Fluorescence microscopy

Fluorescence microscopy was performed basically as described before [20]. Cells were grown statically at 37 °C in C + Y medium, and the expression of the GFP fusion proteins was induced by adding desired concentration of ZnSO_4_. To stain the unfixed cells with fluorescently labelled vancomycin (VanFL) (Molecular Probes) the pneumococcal cultures were grown to OD_600_ 0.2 in C + Y medium, and the samples were labelled with 0.1 μg ml^−1^ of Van-FL/vancomycin (50:50) mixture for 5 min at 37 °C before examination. A quantity of 2 μl of the culture was spotted onto a microscope slide and covered with a 1 % PBS agarose slab. The samples were observed using an Olympus Cell^R^ IX 81 microscope equipped with an Olympus FV2T Digital B/W Fireware Camera and 100× oil immersion objective (N.A. 1.3) (phase contrast). The images were modified for publication using Cell^R^ Version 2.0 software, ImageJ (http://rsb.info.nih.gov/ij/) and CorelDRAW X7 (Corel Corporation). Fluorescence intensity line scans were acquired using ImageJ and plotted as a function of cell length measured with MicrobeTracker Suite [53].

### Cell size analysis

The phase-contrast images were analyzed using automated MicrobeTracker software [53] and cell size parameters were evaluated by the Mann-Whitney rank sum test and plotted using GraphPad Prism 3.0. *P* < 0.0001 was considered as statistically significant. Cell size throughout the text is indicated as the median cell size ± median absolute deviation (MAD).

## Additional file

Additional file 1:
**Table S1.** Oligonucleotides used in this study. (DOCX 15 kb)

## Abbreviations

2D SDS-PAGE: Two-dimensional sodium dodecyl sulfate polyacrylamide gel electrophoresis
CDD: Conserved domain database
CSP: Competence-stimulating peptide
ESTK: Eukaryotic-type serine/threonine protein kinases
ESTP: Eukaryotic-type serine/threonine phosphatases
GFP: Green fluorescent protein
Jag_N: Jag N-terminal domain
Jag_Spn_: *S. pneumoniae* Jag
KEGG: Kyoto encyclopedia of genes and genomes
KH: Ribonucleoprotein K homology domain
MAD: Median absolute deviation
MALDI-TOF: Matrix-assisted laser desorption/ionization- time of flight mass spectrometry
P35: Protein 35 kDa
P40: Protein 40 kDa
PASTA: Penicillin-binding protein and Ser/Thr kinase-associated
PBP: Penicillin-binding protein
PG: Peptidoglycan
PP2C: Protein phosphatase 2C
PPM: Protein phosphatases Mg2+/Mn2+ dependent
pThr: phospho-threonine
P_Zn_: Zinc-inducible promoter
qRT-PCR: quantitative real-time polymerase chain reaction
R3H: Arginine-x-x-x-Histidine domain
SD: Standard deviation
TFE: Trifluorethanol
VanFL: Fluorescently labeled vancomycin
WT: Wild type
