# Supplementary material for: Characterization of pneumococcal Ser/Thr protein phosphatase phpP mutant and identification of a novel PhpP substrate, putative RNA binding protein Jag
Source: BMC Microbiol. 2016 Oct 24;16:247. doi: 10.1186/s12866-016-0865-6 (PMC5078927; doi:10.1186/s12866-016-0865-6)
Supplement: Additional file 1: — Table S1. Oligonucleotides used in this study. (DOCX 15 kb) [file 12866_2016_865_MOESM1_ESM.docx]

| **Additional file 1: Table S1. Oligonucleotides used in this study.** | |
| --- | --- |
| ***Oligonucleotide*** | **Sequence 5´→ 3´ (restriction site underlined)** |
|  |  |
| *AU57* | ATACGCAGAGATGGAGAAAAAT |
| *AU58* | CATTATCCATTAAAAATCAAACGGTACCAGATTCCTCCTTATTTATT |
| *AU59* | AAGCATAAGGAAAGGGGCCCGTAAAATCAGGTTTATCCTGATT |
| *AU60* | ACATATACCCTACTGTTGCTTT |
| *AU67 (SpeI)* | CGCGACTAGTGAAATTTCATTATTAACAGATG |
| *AU68 (NotI)* | CGAAGCGGCCGCTTATCATTCTGCATCCTCCTCGTTCATAGAAAC |
| *AU69* | TCATGTTGGTCAAGCCGGCACTATTGAGCAAGAGA |
| *AU70* | TCTCTTGCTCAATAGTGCCGGCTTGACCAACATGA |
| *AU74* | AATCAGGATAAACCTGATTTTACTACCAGATTCCTCCTTATTTATT |
| *AU75* | GTAAAATCAGGTTTATCCTGATT |
| *AU76* | TTGAACCTGTAAATACTACCACATTTGCCTCCTTAAGATCCGC |
| *AU77* | GTGGTAGTATTTACAGGTTCAA |
| *AU79 (NotI)* | ATTAGCGGCCGCTTACTTATCGTCGTCATCCTTGTAATCTTCTGTATCTACAACAACATAG |
| *AU80* | CCAAGGTCAACGGCTTCTTCACTAACCGTCTTCACA |
| *AU81* | TGTGAAGACGGTTAGTGAAGAAGCCGTTGACCTTGG |
| *JG19* | AAGGAGGCAAATATGGAAATTTCATTATTAACAGATGT |
| *JG20 (NotI)* | ATTAGCGGCCGCTCATTCTGCATCCTCCTCGT |
| *JG21* | TAATAATGAAATTTCCATATTTGCCTCCTTAAGATCCG |
| *JG24* | GATCATGCTGTGGTCAATGAA |
| *JG25* | CATTATCCATTAAAAATCAAACGGCGTTTCTGACCAACATCTGTT |
| *JG26* | AAGCATAAGGAAAGGGGCCCGACAACATTACGGTTGCCCTTG |
| *JG27* | AGTTTCCTCTGTCACAGCCT |
| *JG28* | CCGTTTGATTTTTAATGGATAATG |
| *JG29* | GGGCCCCTTTCCTTATGCTT |
| *JG30* | GACAACATTACGGTTGCCCTTG |
| *JG31* | CAAGGGCAACCGTAATGTTGTCCGTTTCTGACCAACATCTGTT |
| *JG57* | GAAATAGAGTACGATAATGAT |
| *JG58* | CATTATCCATTAAAAATCAAACGGAAAGTCTTGTCCTTTATTTCT |
| *JG59* | AAGCATAAGGAAAGGGGCCCGAAGAAGAGCCTCTGCTCAT |
| *JG60* | TGTGATAGTAAATGGAGTCGT |
| *JG61* | GAAGAAGAGCCTCTGCTCAT |
| *JG62* | ATGAGCAGAGGCTCTTCTTCAAAGTCTTGTCCTTTATTTCT |
| *JG65* | ATGGAAATTTCATTATTAACAGATG |
| *JG66* | TCATTCTGCATCCTCCTCGTT |
| *JG67* | AACGAGGAGGATGCAGAATGA |
| *JG68* | CATCTGTTAATAATGAAATTTCCAT |
| *LN123* | TTCGCTACTTGGAGCCACTAT |
| *LN155 (NotI)* | CGGCGCGGCCGCTTAGTAGTCCAAGTCATCCG |
| *LN214 (BamHI)* | GTGCGTGGATCCGATGATATCAAAGACAGATTGAAA |
| *LN215* | ATTCGAAAATTCTCCTTCTTTCTA |
| *LN218* | TAGAAAGAAGGAGAATTTTCGAATATGCCAATTACATCATTAGAAATA |
| *LN229 (SalI)* | CGGCGTCGACCTACTTATCGTCGTCATCCTTGTAATCCTTCTGGTTCTTCATACATTG |
| *LN231 (EcoRI)* | GTGCGTGAATTCGATGATATCAAAGACAGATTGAAA |
| *NS1* | CTTATCGTCGTCATCCTTGTAATCAAGATGTTTCATATTTGCCTC |
| *NS2* | GATTACAAGGATGACGACGATAAGAGTAAAAAAAGACGAAATCGT |
| *NS3* | GGAGAATTTTCGAATATGGATTACAAGGATGACGACGAT |
| *NS4 (SalI)* | CGGCGTCGACTTAGTAGTCCAAGTCATCCGC |
